# Using genetics to understand biology

**DOI:** 10.1038/s41437-019-0209-z

**Published:** 2019-06-12

**Authors:** Paul Nurse, Jacqueline Hayles

**Affiliations:** The Francis Crick Institute, 1, Midland Road, London, NW1 1AT UK

**Keywords:** Genetic techniques, Eukaryote, Genetic techniques, Eukaryote

The 100th Anniversary of the Genetics Society is a time to celebrate how much this distinguished and congenial Society has contributed to successive generations of geneticists at all stages of their research careers. It is also a time to celebrate genetics itself, a discipline that is both powerful and elegant, and that has provided much insight into the nature of life and how it works.

There are three important pillars of genetics, which permeate all aspects of our understanding of how living organisms function and evolve, and how the processes of life can be investigated. The first pillar is transmission genetics, the basis of heredity. Central to this pillar is the concept of the gene, first proposed by Mendel based on his brilliant abstract analysis and experimentation and championed by William Bateson (1901), even if Fisher’s (1936) subsequent statistical analysis suggested that the experimental data were perhaps just too good. The second pillar is how an organism’s genotype determines its phenotype. This is a problem of how information stored in the gene influences the phenotypic characteristics of an organism. This is essentially a coding problem as suggested by Watson and Crick (1953), who with their exceptional insight realised that the DNA making up the genes could act as, what we would now call, a digital information storage device. DNA sequences determine protein sequences and thus the structure and properties of the proteins that are responsible for phenotype. Combining these two pillars is informative about how living organisms work and how they come about during evolution. The third pillar is concerned with how genetics can be used to investigate the processes underpinning life.

It is this third pillar that is the subject of this article. Genetics methodologies provide powerful ways to investigate biological processes, and can ultimately reveal the underlying molecular mechanisms involved even when there is no knowledge at the outset of a study as to the mechanistic basis of a biological phenomenon. Our discussion will cover in general terms how genetics can be used to investigate how living organisms work, but for practical examples it draws primarily on work from the fission yeast *Schizosaccharomyces pombe*, based on our studies of the eukaryotic cell cycle. In Boxes throughout the text we describe in a more anecdotal way how some of these discoveries were made. The fission yeast was developed as a model organism in the 1950s and 1960s by Urs Leupold and Murdoch Mitchison (Hoffman et al. 2015), two outstanding scientists and generous advisors to PN.

So how has genetics helped to unravel processes and phenomena central to biology? Knowledge of the basis of heredity was extended beyond Mendel particularly by Bateson (1905) using the sweet pea, *Lathyrus odoratus* and Morgan (1910) and his colleagues (1915) using the fruit fly *Drosophila melanogaster*. Major understanding of the generation of phenotype from genotype came from Beadle and Tatum with their research into the fungus *Neurospora crassa*, work that led to the formulation of the one gene one enzyme hypothesis (Beadle and Tatum 1941). Principles of animal and plant development were established by research in many different organisms, but especially important were *Drosophila* (Morgan 1910; Morgan et al. 1915), the nematode worm *Caenorhabditis elegans*, developed as a model genetic organism by Brenner (1972), and the cress weed *Arabidopsis thaliana* (Feenstra 1964). A beautiful example of the power of developmental genetics are the studies in *Drosophila* of homeotic genes that when mutated can alter developmental fate, such as changing whether a leg or an antenna is formed in a particular location on the fly (Lewis 1978; Struhl 1981). Understanding the logical basis of gene regulation was explored by Jacob and Monod (1961) using the bacterium *Escherichia coli*, whereas understanding of neural development has drawn heavily on studies in *Caenorhabitis* (Bargmann 1998) as well as of the mouse, *Mus musculus* (Ellenbroek and Youn 2016). Mechanisms for a variety of eukaryotic cell biological phenomena have been revealed by research in the yeasts, including regulation of the cell cycle (Hartwell et al. 1970; 1973; Nasmyth and Nurse 1981; Nurse et al. 1976), secretion (Novick and Schekman 1979) and autophagy (Takeshige et al. 1992; Thumm et al. 1994).

These are just a few examples of the many processes illuminated by the application of genetic analyses. Most of these studies have been carried out with model genetic organisms characterised by ease of manipulation, short-generation times, ability to be mated, mutagenized and screened, and a suitability for molecular genetics. The topic of model organisms amenable to genetic analysis is covered more fully by Jonathan Hodgkin elsewhere in this volume.

## Genetic screens

Forward genetic screens are usually the starting point for the genetic investigation of biological phenomena. The principle behind this type of screen is to search for mutants that will be informative about the process of interest. This requires procedures that allow efficient mutagenesis, easy screening, and the rapid detection of recessive mutations, either by using haploid and hemizygous organisms or via homozygosis of mutations in diploids. However, the very first step needs to be an act of creative imagination. What mutant phenotype can be imagined that will be informative about the process under study? This is crucial if the subsequent mutant screen is to be successful. A mutant screen is an exercise in pathology, a hunt for mutants with abnormal behaviours that disturb the process but at the same time are revealing about the normal functioning of that process. To make good choices about an appropriate mutant screen requires good knowledge about the biology of the model organism being used. As Barbara McClintock aptly put it, what is needed is a ‘feeling for the organism’ (pers com. PN). Genetics is derided by some for being too reductionist, but in fact the opposite is true; successful genetic studies need a good understanding of how the biological entity under investigation behaves as a whole, be it a cell, an organ, or an organism.

**Fig. 1:**
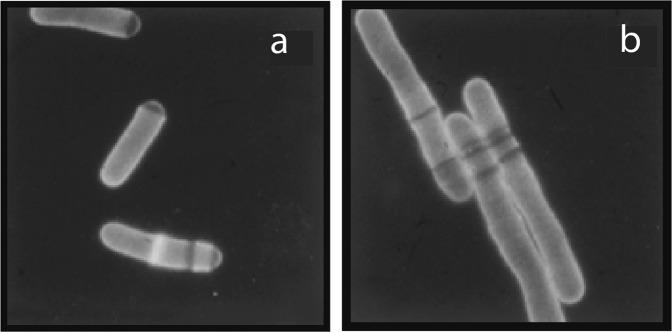
Cell cycle (*cdc*) mutant. **a** A *cdc*^ts^ mutant at the permissive temperature. Cells can grow and divide and do not elongate. **b** A *cdc*^ts^ mutant at the restrictive temperature is unable to divide but continues to grow and so has an elongated cell phenotype. The cell wall and septum are stained with calcofluor

The use of forward genetic screens in the fission yeast *S. pombe* for study of the cell cycle and its control, provides a useful case study. Fission yeast is a single-celled haploid eukaryotic organism, a rod-shaped cylinder that grows by cell elongation at the tips (Fig. 1a). At the beginning of their cell cycle, wild type cells undergo a short G1 followed by S-phase. A subsequent long G2 ends with mitosis and is followed by cytokinesis, where a centrally placed septum divides the cell into two equally sized daughter cells. For genetic studies of the cell cycle, mutants need to be identified that are unable to complete the cell cycle successfully, and thus cannot undergo cell division. Such mutants will be lethal in a haploid organism, so the mutant phenotype needs to be conditional, which is the failure to complete cell division only in certain restrictive conditions allowing the cells to be kept alive when grown in the permissive conditions. The approach used was to mutagenize haploid cells to generate mutations, and then to screen for mutants that had a temperature sensitive phenotype because they had a thermolabile protein that was dysfunctional at high temperature.

But what phenotypes should be screened for that are relevant to the cell cycle? The search is for mutant cells that cannot divide, but the problem is that there are many ways of stopping a cell from dividing, most of which are not informative because they are not directly involved in the cell cycle. Any gene required for the growth of the cell which is rendered non-functional will also block cell division if made non-functional by mutation. This includes defects in protein, RNA and other macromolecular synthesis, as well as metabolism and energy production, in fact most of the functions needed for the life of the cell. This problem can be overcome by selecting temperature sensitive mutants, which cannot complete the cell cycle but that are still able to grow because activities required for growth are still taking place. These rod-shaped fission yeast cells continue to grow but do not divide and thus form elongated cells which can be identified by microscopic visual screening (Fig. 1) (Nurse et al. 1976). This visual screen was carried out, and the first search isolated 27 mutants, called *cdc* for cell division cycle, which were found to define a total of 14 genes in complementation tests (Nurse et al. 1976). A second similar screen isolated 59 mutants defining a total of 10 further genes (Nasmyth and Nurse 1981). These genes formed the basis of initial work on the cell cycle, showing that well designed forward genetic screens can identify genes required for a biological process of interest and open it up for study.

## The unexpected phenotype

The random forward genetic screens also have the potential to bring about serendipitous discovery, and designing screens that are somewhat open ended can assist such chance outcomes. Visual screens of the type used to find cell cycle mutants in fission yeast provide a chance to discover mutants that are different from what was originally conceived. In one sense, such open-ended searches allow ‘nature’ to deliver unexpected mutant phenotypes to any geneticist ready to recognise such opportunities.

Such serendipity had a hand in uncovering the role of the *cdc2* gene at the G1–S transition. At the time, *cdc2*^ts^ mutants were thought only to arrest at the G2–M transition and were used as a negative control for a screen to identify *cdc*^ts^ mutants that were blocked in G1 before commitment to the mitotic cell cycle and thus could still undergo sporulation. The *cdc2*^ts^ mutant consistently showed a low level of sporulation at the restrictive temperature. Further experiments showed that in fact Cdc2 function was also required for the G1–S transition (Nurse and Bissett 1981). The majority of *cdc2*^ts^ cells were blocking in G2 with a small percentage blocking in G1, and it was these G1 cells that were able to undergo conjugation and sporulation because they were blocked before commitment to the cell cycle. Demonstrating that a single-gene function was required at two completely different control points in the cell cycle was a significant step forward. (Box 1—Believing data).

**Fig. 2:**
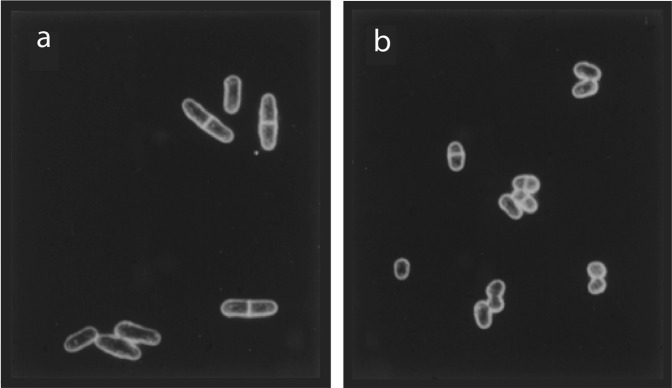
*Wee* mutants. **a** Wild-type cells. **b** *Wee* mutant cells that are advanced into mitosis and divide at a small cell size. The cell wall and septum are revealed by dark field microscopy

Another example of chance discovery was the finding of a micro-colony of small cells during a screen for elongated cell cycle mutants (Box 2—Serendipity). Seeing such cells led to the realisation that they were being advanced into mitosis and cell division, before they grew to the normal size for entry into mitosis. This small cell phenotype (Fig. 2) revealed that there were rate-limiting steps acting in the cell cycle, one of which controlled the timing of the G2 to mitosis transition, providing new insight into cell cycle control. Following this chance observation, a systematic screen for small cell mutants was carried out (Thuriaux et al. 1978). The mutants were called *wee* (meaning small in Scotland) because they were first observed in Edinburgh. Two genes were identified, *wee1* and *cdc2* (originally called *wee2*), now known to encode CDK1, which is the name for *cdc2* orthologues in all eukaryotic organisms. (Box 3—Throwing mutants away). Wee1 acts negatively and Cdc2 positively at the G2–M transition (Nurse and Thuriaux 1980). The *wee* phenotype of *cdc2* was a consequence of a gain of function mutation, which would not have been found by screening a genome wide deletion collection (described in the next section).

**Fig. 3:**
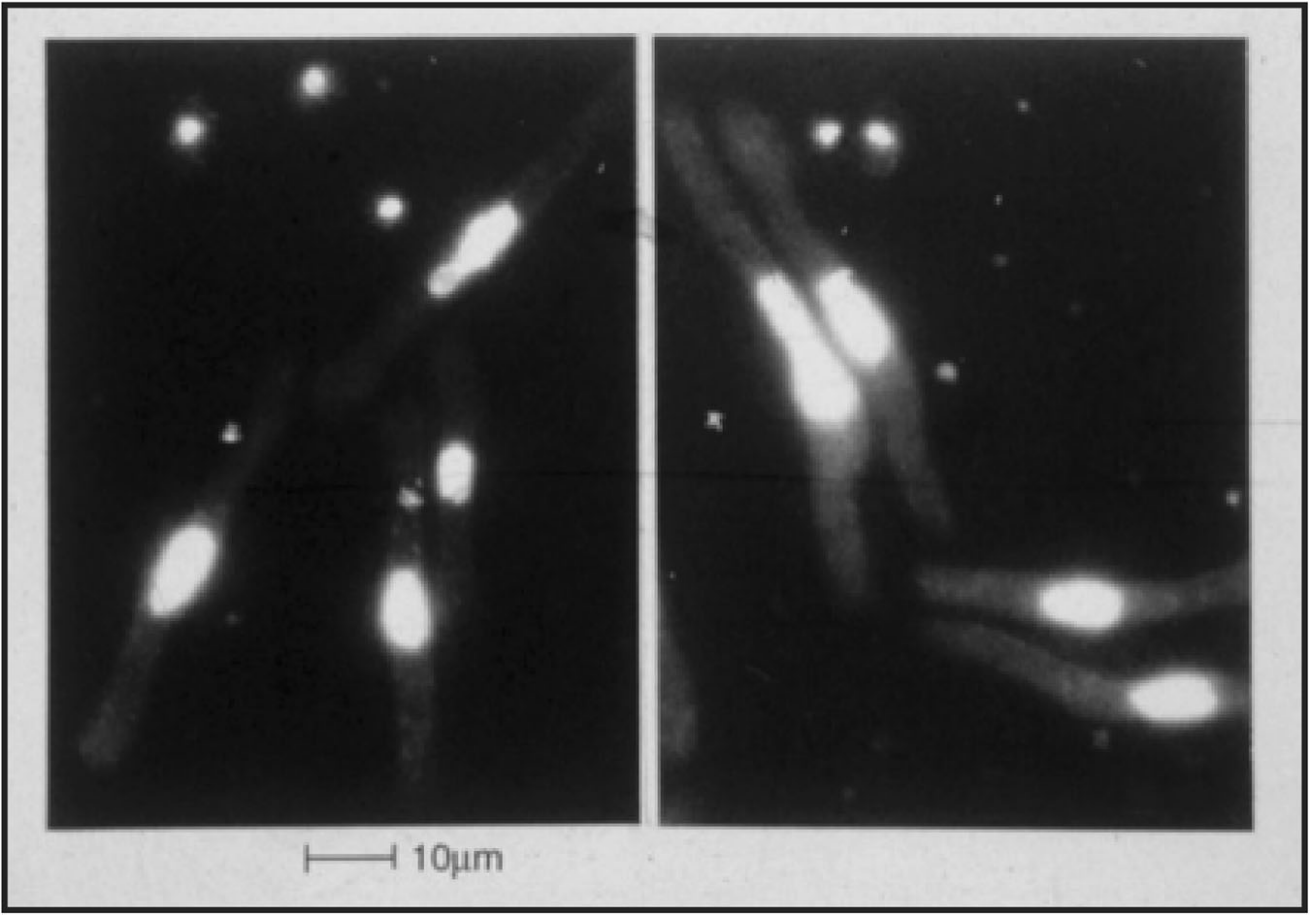
Cells undergoing DNA re-replication in the absence of mitosis. Cells undergoing repeated rounds of DNA replication in the absence of mitosis produce elongated cells with increased ploidy. The nuclei are stained with the DNA-specific dye DAPI

Once the logic of advancement through the cell cycle was revealed as useful for understanding cell cycle control, it could be applied to other cell cycle events, such as the control of S-phase. Screens to identify mutants that could advance cells into S-phase produced mutant cells with a very unexpected phenotype- elongated cells with huge nuclei that had a high DNA content (Fig. 3). The phenotype was caused by overexpression of either *cdc18* (*cdc6* in other organisms) or *rum1*, advancing cells into S-phase and leading to DNA re-replication and thus higher ploidy (Moreno and Nurse 1994; Nishitani and Nurse 1995). These genes and another gene *cdt1* (Hofmann and Beach 1994) were found to be core to the control acting over the onset of S-phase (Nishitani et al. 2000; Yanow et al. 2001). A deletion of the cyclin B *cdc13* gene also caused this phenotype and it was subsequently shown that the Cdc13—CDK1 complex, required for entry into mitosis, was also required to prevent a further round of DNA replication from taking place from G2, thus ensuring that there is only one S-phase each cell cycle (Hayles et al. 1994).

Serendipitous discoveries such as these have been found in many different organisms and can be extremely useful in opening up new understanding of biological phenomena.

Box 1 Believing data The approach being used to define the point of commitment in the cell cycle had been developed by Lee Hartwell (1970) working with budding yeast. His idea was to block cell cycle progression with temperature sensitive *cdc* mutants and challenge these various cell cycle blocked cells to conjugate. If they were ‘uncommitted’ to the cell cycle they would be able to conjugate but if ‘committed’, that is past a commitment point in the cell cycle called ‘start’, they would not be able to conjugate. The data outcome should have been binary for this experiment, which is 0% if committed (in practice 0–5%), and 100% if uncommitted (in practice 80–100%). This worked well for all the *cdc* mutants tested, except for *cdc2*, which gave around 20% conjugation. The experiment was repeated many times to try and get the ‘right answer’, which should have been 0.5% as *cdc2* mutant cells were thought to block in G2. But that result was never obtained, it was always ~ 20%. Only after several months did PN wonder whether 20% might in fact be the right answer, and if that was the case what did that result mean? After a few days thinking, an explanation popped up. The experimental results could be explained if the *cdc2*^ts^ mutant was blocking at two points in the cell cycle, at G1 before start and later in G2 at the mitotic control. This turned out to be correct, and was the first demonstration that CDKs operate at the two major control points in the cell cycle. Believing data rather than wanting the ‘right result’ can pay off.

Box 2 Serendipity The small cell-sized *wee* mutants were discovered entirely by chance. A visual screen was being carried out by PN looking for elongated cells in micro-colonies, which had formed after cells had been centrifuged through a density gradient to enrich large cells. The objective of this screen was to identify new elongated conventional *cdc* mutants. During this visual screen the exact opposite was found, a micro-colony of small cells. These *wee* mutant cells tend to clump together, probably explaining why they turned up where they did in the density gradient. It was only when these cells were spotted that it became obvious that if cells are advanced prematurely through the cell cycle (thus altered in a rate-limiting control step of cell cycle progression), that they will divide faster than they can grow and as a consequence will divide at a small size. Obvious, of course, in hindsight but rather less so beforehand, and all owing to serendipity.

Box 3 Throwing mutants away This gain of function *wee* mutant should never have been isolated. The screen for new *wee* mutants was extremely laborious, yielding only 1–2 mutants every week. The goal PN set was to isolate 50 such mutants and this took the best part of a year. The *wee2* mutant was dominant, because it resulted in a gain of function. It was isolated towards the end of the study, after every mutant isolated up to that time had been found to be an allele of *wee1*. The *wee2* mutant isolate was spotted late on a rainy Friday afternoon on a plate very contaminated with a fungus. Looking too difficult to purify from the fungal contamination and given it was likely to be yet another allele of *wee1* (like the previous 47 or so mutants), the plate and the mutant were thrown away in the rubbish bin. Later that evening PN felt guilty, and cycled back to the laboratory in the rainy cold Edinburgh weather retrieved the discarded plate and eventually purified the new mutant. This was the only *wee* mutant that was not an allele of *wee1*, and defined a second gene *wee2* that was eventually shown to map within *cdc2*.

## Systematic genomic screens

Forward mutagenesis based on the screens described above have proved to be very informative about a process but are neither systematic nor comprehensive. In contrast, systematic genome -wide screens allow the identification of a more complete catalogue of gene functions that are involved in the biological process of interest. Such screens are usually based on genetic approaches that eliminate or downregulate gene functions, so by definition will only identify genes that generate the mutant phenotype when they lose or reduce function. This is a limitation because gain of function mutants can be very illuminating, but this shortcoming is offset by the comprehensive nature of the screen. Molecular genetics can be used to systematically delete gene functions on a genome -wide basis using homologous recombination, an approach that has been valuable with bacteria (Baba et al. 2006) and the yeasts (Baudin et al. 1993; Giaever et al. 2002; Kim et al. 2010; Winzeler et al. 1999) and to some extent with multicellular organisms (Frokjaer-Jensen et al. 2010; Gong and Golic 2004). Alternative approaches include genome -wide systematic reduction of gene expression through RNAi knockdowns (Dietzl et al. 2007; Kamath and Ahringer 2003; Kiger et al. 2003) and overexpression screens that are useful for drug target screening (Arnoldo et al. 2014). CRISPR-Cas is also a useful gene-eliminating methodology, particularly for multicellular organisms (Dickinson and Goldstein 2016; Doudna and Charpentier 2014; Gratz et al. 2015). Its use and that of similar approaches will allow analysis of genome -wide gene deletions to be carried out in organisms that are less amenable to genetic studies.

In the fission yeast, a genome-wide gene deletion collection was constructed using homologous recombination to delete 4836 (98.4%) of the 4914 protein coding genes annotated at the time (Kim et al. 2010). Essential gene deletions are maintained as heterozygous deletion diploids, and the haploid deletions can be derived from each of these diploids by sporulation followed by germination of the haploid spores. Of the 4836 deletions constructed, only 26% were found to be essential for cell viability under the growth conditions used. By visual screening, all gene deletions after germination, it was possible to identify the genes that caused cell elongation when deleted because they were unable to complete the cell cycle (Hayles et al. 2013). A total of 513 cell cycle genes were identified of which 341 *cdc* genes were essential, more than double the number previously identified, despite the fact that earlier conventional forward genetic screens had been carried out for over 40 years. Interestingly, it was mainly the previously identified genes that showed the strongest *cdc* phenotype. A further 172 gene deletion strains were identified, which are not essential for viability but are elongated at cell division, and therefore are delayed in completing the cell cycle, and so contribute to cell cycle progression. (Box 4—I am not a robot). A screen of ~ 3000 of the non-essential gene deletions for cells that divided at a small size, and are thus defective in the timing of the G2–M transition (Navarro and Nurse 2012) identified 18 genes that are likely to be rate-limiting for cell cycle progression (see—Understanding the networks).

These systematic genomic approaches have generated an almost complete catalogue of the genes required for successful cell division, identifying the majority of genes that need to be considered when thinking about the eukaryotic cell cycle and particularly control of the cell cycle. From this ‘naming of the parts’ exercise for the cell cycle, we concluded that ~ 10% of fission yeast genes have roles in the cell cycle.

Systematic genomic screens similar to this have been carried out using model organisms to study many other biological processes, for example, identifying genes affecting cell morphology in *Drosophila* (Kiger et al. 2003) or UV sensitivity in budding yeast (Birrell et al. 2001). Given the conservation of molecular mechanisms throughout the living world, work on model organisms like bacteria, yeasts, worms and flies is likely to be relevant to all eukaryotes including ourselves.

Box 4 ‘I am not a robot’ Many genome-wide screens are carried out using robots and image analysis. This fission screen was less sophisticated and carried out by a single human operator JH because this allowed more subtle or unexpected mutant phenotypes to be detected which may not be observed using automated procedures. It did not require extensive upfront development of techniques, which can be time consuming and sometimes distracting, but did require a good understanding of the organism and what the different phenotypes may mean. This approach resulted in a highly effective screen, which generated a robust collection of cell cycle mutants. There can be advantages in ‘not being a robot’.

## Becoming molecular and cellular

**Fig. 4:**
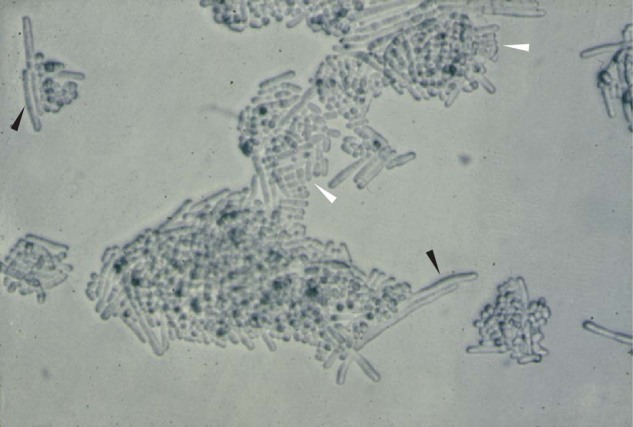
Cloning by complementation. A *cdc2*^ts^ mutant is able to grow at the restrictive temperature when cells carry the human CDC2 gene on a plasmid. This gene is able to complement the yeast *cdc2*^ts^ mutant function and cells can grow and divide to form colonies (white arrow heads). Cells that lose the plasmid are no longer able to divide, but continue to grow and form elongated cells (black arrow heads)

Genetics is generally rather abstract in the ways in which it reveals how things work. Biological phenomena and processes are described in terms of gene names, but do not provide mechanistic explanations that describe the nature of the molecules and the biochemical processes involved. To move from abstract explanations to biochemical mechanisms requires cloning the relevant genes. This is possible by genetically mapping the genes and determining their position within the genome followed by sequencing of the region, a methodology greatly helped by the availability of whole-genome sequences. Another approach can be used when efficient DNA transformation procedures are available. Gene libraries can be constructed and transformed into mutant cells to select for clones that rescue the mutant function. This is cloning by complementation (Fig. 4), and was the approach used to clone genes in the yeasts (Beach et al. 1982; Nasmyth and Reed 1980). (Box 5—Is it a contaminant?).

In fission yeast complementation, cloning was combined with whole genome sequencing and positional mapping to generate the sequences of the majority of cell cycle genes (Kohli et al. 1977; Wood et al. 2002) (Box 6—Cottage Industry). With the availability of gene sequences, it is possible to predict their putative molecular functions. Biochemical investigations of these molecular functions are facilitated by purification of the gene products; for example, through tagging the genes or raising antibodies via protein expression in bacteria and protein purification, or by peptide synthesis. With gene product purification, comes the ability to perform biochemical assays, providing the link between genetics and molecular mechanism. Using tagged genes or specific antibodies against gene products also allows the cellular locations and behaviour of gene products of interest to be determined. Many cell cycle genes in different organisms have been tagged, and the locations and levels of the tagged proteins have been monitored as cells proceed through the cell cycle. Combining molecular and cellular information leads to the development of mechanistic explanations of biological processes, linking molecules to phenotypes.

One of the advantages of toggling between genetic, cellular and molecular data is that it increases the robustness of explanations. Each of these spheres of investigation have strengths and weaknesses that can complement each other, generating different types of explanations both abstract and mechanistic, thus strengthening the understanding of biological phenomena and processes. This is considered further in the next section.

Box 5 Is it a contaminant? The ability to transform fission yeast with exogenous DNA was needed to clone cell cycle genes by complementation (Beach and Nurse 1981). It was developed in the laboratory about a year or two after the technique had been shown to work for budding yeast (Beggs 1978). Initial trials were based on making protoplasts, which had to be plated after suspension in an osmoticum contained within soft agar. Unfortunately, the wrong soft agar was used by PN, which led to partial solidification in the tubes before plating. Only by shaking out the setting agar and squashing it down in the plate with the plate lid could the experiment be completed. The outcome was a complete mess prone to contamination, and the whole experiment should have been thrown away. However, the plates were put in the incubator ‘just in case’. Amazingly colonies grew up within the shattered agar lumps although they could not be examined microscopically. It was assumed that these were contaminants, but in fact they were transformed fission yeast, the first ever to have been made.

Box 6 Cottage industry Fission yeast was never on the ‘hot-list’ for the genomic sequencing community, unlike budding yeast, the worm, and the fly, for example, and no funding could be raised to get the organism sequenced. Luckily, PN met Bart Barrell who had worked with Fred Sanger, and Bart had resources from a funding agency to contribute to the sequencing of budding yeast. BB had rather too much support for the budding yeast sequencing, so he and PN cooked up the idea of using the excess funding to sequence fission yeast. Would the funding agency notice? Unfortunately they did! About half the genome sequence was done in 6 months but when we went to the funding agency to get the rest of the money to make fission yeast the second eukaryote to be fully sequenced the agency was not amused, and did not provide the extra resources. This meant we had to go to the EU and fund about a dozen laboratories around Europe as a cottage industry to finish the sequence. This took over a year more, but the sequence was completed and completed to a high standard, which was not surprisingly given BB’s high standards and pedigree. Fission yeast ended up being the 4th free-living eukaryote to be fully sequenced.

## Describing the networks

The next stage in understanding is to generate the networks of interacting molecules, the interactome, which is responsible for the proper functioning of the biological processes. This requires comprehensive databases, examples being pombase.org, flybase.org, wormbase.org, yeastgenome.org and thebiogrid.org (Chervitz et al. 1999; Gramates et al. 2017; Howe et al. 2016; Lock et al. 2018; Stark et al. 2006). These databases contain a complete list or near-complete list of the genes together with gene product and genetic interactions derived from forward genetic and systematic genomic screens and biochemical analyses. The components encoded by these genes can be organised together to generate a network underlying the process under study, using programmes such as esyn.org (Bean et al. 2014) or string-db.org (Jensen et al. 2009). Two types of data are available to generate these networks, based on either physical interactions using techniques such as yeast two hybrid analysis, affinity pull downs, and mass spectroscopy, or genetic interactions revealed by analyses such as suppression, synergism and epistasis. These physical and genetic methodologies complement each other because of the conceptually different approaches they use, so there is extra confidence when similar conclusions are reached.

Once an interactome underpinning a biological process has been generated there can be a tendency amongst researchers to leave it there, although such descriptions of networks are usually insufficient for a proper understanding of how the process works. Having the protein sequences derived from the gene sequences available allows predictions of the biochemical functions associated with the gene products to be made. From these predictions, at least a partial understanding of the network of biochemical processes can be built up. But what really is required is to move from identifying the components and their biochemical activities, to comprehension of the basic principles and operational logic that are critical to the network of interest. This is difficult because there is no clear investigative pathway to follow, but we shall consider what is useful when tackling this problem.

## Understanding the networks

A major question is what types of explanations lead to a meaningful understanding of the biological process or phenomenon of interest? One concept we have found useful is to consider the process under study in terms of the management of information, because this can help in moving from chemical description to biological understanding. Issues pertinent to the management of information are inputs of information into a process, the integration, processing and storage of information intrinsic to the process, and how information determines the subsequent output that bring about a particular outcome for that process. Two classical biological phenomena that illustrate this concept are the structure of DNA and regulation of the *Lac* operon. The structure of DNA describes how the atoms are positioned with respect to each other in the DNA molecule. However, biological understanding only emerges when the management of information is considered. The structure of DNA only made sense biologically when it was shown that it was essentially a digital information storage device (first proposed by Mikhail Neiman (1964)) that could be precisely copied, explaining both coding and the inheritance of information (Brenner et al. 1961; Crick et al. 1961; Leder and Nirenberg 1964; Meselson and Stahl 1958). Similarly, the behaviour of the *Lac* operon can be described in terms of the chemistry of the molecules involved and how they interact with each other to control gene expression. However, biological understanding only comes when it is recognised that information flow through the system results in a negative feedback loop, which regulates the level of the *Lac* operon expression (Jacob and Monod 1961). Cell cycle control in fission yeast has also profited from this thinking. Informational inputs to the cell cycle control acting over mitosis and cell division come from the increasing size of the cell as the cell cycle proceeds, and from monitoring whether the DNA is undamaged and fully replicated. This information is integrated at the level of the cyclin dependent protein kinase (CDK1) activity, and output from the cyclin B–CDK1 complex results in phosphorylation of proteins with key roles at the onset and progression through mitosis (Blethrow et al. 2008; Swaffer et al. 2016).

A second concept that needs to be considered is what is meant by control. Improved biological understanding of a process often comes from knowing how the controls operate in the process. Sometimes the term control is used rather loosely, for example, when it is thought that control is associated with any step that is necessary for a process to work, even though with such a view nearly all steps can be considered as controls. It is more useful to identify the major rate-limiting steps that contribute in a significant way to the rate at which a biological process occurs. Thinking about this with respect to the cell cycle had its origins with discussions about rate-limiting steps in metabolic pathways, which revealed that rate-limiting controls can be distributed among a number of different steps in a network (Kacser and Burns 1995). It is also important to realise that the steps which are rate limiting can change depending on the biological context. An experiment useful for thinking about rate-limiting steps is to undertake ‘local perturbation analysis’. This approach requires the rate of an individual step in a network to be varied by a small amount, and then for the consequences of that local perturbation on the rate of the overall process to be determined. A systematic way to undertake local perturbation experiments is to use a haploinsufficient approach. Studying genes of interest in a heterozygous deletion situation where the level of component is likely to be reduced by half, and to test how this affects the process of interest. Over 500 cell cycle genes of fission yeast were investigated to identify those that delayed or advanced cells into mitosis. This led to the identification of 17 haploinsufficient genes that have impacts on the overall progression through the cell cycle (Moris et al. 2016). The reasoning behind this analysis was that mutants delaying or advancing the rate at which cells proceeded through the cell cycle, would identify potential control points in the cell cycle. This work identified tyrosine phosphorylation of CDK1 as a critical rate-limiting step for the timing of entry into mitosis. This approach together with a screen of the non-essential deletion collection for *wee* mutants (Navarro and Nurse 2012) has identified a set of 33 genes whose activities are likely to be rate limiting for cell cycle progression.

A further way is to think about controls is as decision steps, such as commitment to a specific developmental fate or entry into the cell cycle. A biological decision is made within the network that leads to the process either taking place or not. An extension of this idea is the checkpoint control by which a cell determines whether it should inhibit or continue with a process (Hartwell and Weinert 1989). Understanding this has been important for cell cycle studies, particularly when applied to controls acting over the onset of mitosis when DNA replication is incomplete or DNA is damaged (al-Khodairy and Carr 1992). These checkpoint inputs were found to operate in fission yeast through inhibitory tyrosine phosphorylation of CDK1 (Rhind et al. 1997).

Concepts such as these are useful for giving biological meaning to an understanding of how a network brings about a particular process. To test these ideas further needs knowledge of the molecular steps in the network and the context of how they operate in the cell or organism. It requires detailed hypothesis testing, and experimentation that combines genetics, biochemistry and cell biology. As data accumulate, it should be possible to develop systematic, theoretical and in silico approaches. Knowledge of the biochemical activities associated with different steps in the network and how they interact with each other can be combined with knowledge of whether these combinations of activities generate logical modules critical to network operation. For example, GTPases and their associated regulators can act as switches, amplifiers and timers within a network. Extending such knowledge to the various component combinations that make up networks may assist working out how they operate (Karlebach and Shamir 2008).

Perhaps a more radical approach to understanding the networks underpinning a biological process or phenomenon is to simplify the network. The thinking here is that systematic screens can identify many of the components that need to be considered when working out how a biological network operates, but they are unlikely to all be of significant importance. As a consequence components can be identified that when removed simplify regulation, while still maintaining the core operations of the network. In principle, this allows attention to be focussed on the key elements necessary to maintain core operations, reducing the risk of being distracted by functions that are more peripheral. This was used in fission yeast cell cycle studies to demonstrate that the four cyclin–CDK1 complexes identified as having roles in the mitotic cell cycle and the six cyclin–CDK1 complexes in the meiotic cell cycle, can all be replaced by a single cyclin B-CDK1 (Coudreuse and Nurse 2010; Gutierrez-Escribano and Nurse 2015). This means that orderly progression through the cell cycle is not driven by a series of qualitatively different cyclin–CDK1s as is generally assumed, but can be brought about by the rising activity of a single monomeric cyclin B–CDK1 as cells proceed through the cell cycle (Swaffer et al. 2016). This approach identified the core principle underlying CDK1 regulation of the cell cycle as being based on controlled quantitative increase and decrease in CDK1 activity.

## Post-script

In this article, we have tried to demonstrate how genetics can help to understand biological processes and phenomena. Key to this are powerful classical and molecular genetic methodologies, imaginative approaches, and the ability to move between genetics, biochemistry and cell biology. For this type of approach to work well, it requires a clear focus on the physiology of the organism under study and for the researcher to have a true ‘feeling for the organism’.
